# Tactical Variables Related to Gaining the Ball in Advanced Zones of the Soccer Pitch: Analysis of Differences Among Elite Teams and the Effect of Contextual Variables

**DOI:** 10.3389/fpsyg.2019.03040

**Published:** 2020-01-21

**Authors:** Javier Fernandez-Navarro, Carlos Ruiz-Ruiz, Asier Zubillaga, Luis Fradua

**Affiliations:** ^1^Department of Physical Education and Sport, Faculty of Sport Sciences, University of Granada, Granada, Spain; ^2^Department of Physical Education and Sport, Faculty of Sport Sciences, University of the Basque Country UPV/EHU, Vitoria-Gasteiz, Spain

**Keywords:** performance analysis, La Liga, association football, defending tactics, match analysis, elite soccer

## Abstract

Attacking tactical variables have been commonly studied in soccer to analyze teams’ performance. However, few studies investigated defensive tactical variables during match-play and the influence of contextual variables on them. The aims of the present study were (1) to examine the defensive behaviors of soccer teams when gaining the ball in advanced zones of the pitch and (2) to evaluate the effect of contextual variables on these defensive behaviors. A sample of 1,095 defensive pieces of play initiated in the opposing half of the pitch obtained from 10 matches of the season 2010/11 of La Liga and involving 13 teams was collected using the semiautomated tracking system Amisco Pro. Five defensive tactical variables, the outcome of defensive pieces of play, and contextual variables (i.e., match status, venue, quality of opposition, and match period) were recorded for every defensive piece initiated in the opposing half of the pitch. Results showed that there were significant differences among teams in the outcome of defensive pieces of play originating from the opposing half (χ^2^ = 111.87, *p* < 0.01, φ_c_ = 0.22), and in the outcome of offensive pieces of play following ball gains (χ^2^ = 49.92, *p* < 0.001, φ_c_ = 0.22). Cluster analysis revealed four groups describing different defensive behaviors from high-pressure to a defense close to their own goal. Match status (χ^2^ = 25.87, *p* < 0.05, φ_c_ = 0.11) and quality of opposition (χ^2^ = 21.19, *p* < 0.05, φ_c_ = 0.10) were the contextual variables that showed a significant effect on defensive pieces of play initiated in the opposite half of the pitch. Teams winning gained more balls in the zone close to their own goal, and losing teams gained more balls in advanced zones of the pitch. Moreover, the greater the quality of the opponent the lesser the chance of gaining the ball in advanced zones of the pitch. Neither venue or match period influenced the defensive pieces of play analyzed. Soccer teams could employ a similar analysis to improve their performance and prepare for opposition teams in competition.

## Introduction

The analysis of tactical behaviors in soccer is an important aspect in elite level and is increasing its attention in research (Sarmento et al., 2014; Rein and Memmert, 2016). Recent studies explored the use of current approaches to evaluate tactical behaviors in soccer (Low et al., 2019). This research examined collective tactical behaviors from positional data and suggested that future studies should focus on contextualizing these positional data and bridging the gap between science and practice by incorporating the needs of the coaching staff. In the same way, Memmert et al. (2017) showed that positional data was useful to develop collective performance indicators in order to understand the dynamics of a soccer match. This study also highlighted that these new approaches can be helpful in sport practice and could help coaches and other practitioners to modify their training methods. In addition, these studies suggested that future studies should explore in more detail the collective attacking and defensive actions in soccer.

Previous research in soccer performance analysis has evolved from a traditional approach with the use of video data to a novel approach using positional data. Memmert and Raabe (2018) determined four different stages that describe the evolution of match analysis; being 1.0 and 2.0 based on video data and considering quantitative assessment and qualitative assessment respectively, and match analysis 3.0 and 4.0 based on positional data with the former considering physiological and technical assessments and the latter considering dynamic tactical assessments. This evolution has been possible mainly, because of the progress of computer science and technology (Perl et al., 2013; Memmert et al., 2017). Hence, research conducted prior to the 4.0 stage exploring the tactical aspects of soccer has focused on the evaluation of performance indicators (REF), such as passes (Hughes and Franks, 2005; Redwood-Brown, 2008), crosses (Lago-Ballesteros and Lago-Peñas, 2010; Pulling et al., 2018), or shots (Ensum et al., 2005). In contrast, modern match analysis approaches use positional data to analyze tactical behavior and dynamics through novel metrics. For instance, passing effectiveness or space control (Rein et al., 2017).

Although there are many studies focusing on attacking behaviors in soccer, some research has also evaluated defensive aspects. For example, Filho et al. (2013) evaluated the defensive behavior of Brazilian and Italian soccer national teams by measuring goals scored, goals conceded, and points per game. Vogelbein et al. (2014) evaluated the time that soccer teams in Bundesliga employ to regain ball possession. This study showed that recovering ball possession quickly after losing the ball was associated with a successful defensive performance. In addition, a previous study examined the recovery patterns of teams playing the 2010 FIFA World Cup (Barreira et al., 2014). This study measured the type of ball recovery (e.g., intervention, tackle, goalkeeper save) and the zones where the ball was regained to assess the subsequent success of attacking play. The results showed that a direct recovery of ball possession in mid-defensive central zones increases the latter attacking efficacy. In the same way, Almeida et al. (2014) measured the type and zone of ball recovery to examine the effect of contextual variables on regaining possession in UEFA Champions League teams. Home and losing teams used to defend in more advanced zones of the pitch and strong teams were more effective when applying defensive pressure in more advanced zones. Moreover, recent research has also used a dynamical systems approach to evaluate unstable game states during a soccer match, integrating defensive actions with the attacking ones; and suggesting that teams handle unstable situations differently (Kim et al., 2019a, b).

In addition to tactical variables, recent research highlighted the importance of styles of play for analyzing soccer performance (Tenga and Larsen, 2003). Styles of play are characteristics playing patterns demonstrated by a team during games (Hewitt et al., 2016), and are useful to describe the general tactical behavior of the team in competition. Previous studies identified different styles of play in the Spanish and English leagues (Fernandez-Navarro et al., 2016; Gollan et al., 2018; Castellano and Pic, 2019), Chinese league (Lago-Peñas et al., 2017), and Greek league (Gomez et al., 2018). These studies showed a majority of attacking styles of play, such as; possession, direct, counterattack, or crossing. On the other hand, some defensive styles of play were identified according to the zone of the pitch where the ball was regained. High pressure, low pressure, pressure on wide areas, and pressure on central areas were the defensive styles of play identified (Fernandez-Navarro et al., 2016). Kempe et al. (2014) showed that successful teams prefer possession play, and similarly, Yi et al. (2019) showed that teams using possession play scored higher for the variables related to goal scoring, attacking and passing. However, according to other studies that explored further the characteristics of the possession style of play, by analyzing the factors that explain the self-organization in possession team play (Chassy, 2013), and examining the success of this possession play (Collet, 2013); the former study showed that possession itself did not predict shooting opportunities, and in the same way, the later demonstrated that possession was a poor predictor of performance when controlling for team quality and home advantage.

However, although measuring styles of play in soccer shows useful information about overall team strategies, the study of specific tactics by teams would provide more detailed insight into their tactical behavior. Gaining the ball in advanced zones of the pitch is a behavior that has been considered in research to evaluate the tactical performance of soccer teams (Almeida et al., 2014), and is also an important aspect for coaches and practitioners. For example, Gomez et al. (2012) measured, among other attacking variables, ball recovers in each zone of the pitch to examine the effects of location and final outcome on them. Their results did not show any effect of those contextual variables on ball recovers in advanced zones but showed effects in other zones of the pitch. Nevertheless, a recent study measured the ball recovery location and the position of the defensive line to examine the effect of match conditions on defensive positioning (Santos et al., 2017). This study demonstrated that ball recovery location increased when the team was losing and the position of the defensive line decreased when playing away and facing strong opposition. This previous research showed the importance of considering the zones of the pitch when evaluating tactical defensive performance, although more variables to describe the gaining of the ball in advanced zones of the pitch should be evaluated to analyze these tactics. Consequently, using other defensive variables such as the distance of defending players to opposition, duration of the defensive actions, or the distance of the last outfield player to the goal line could be useful to describe defensive behavior and the tactic of gaining the ball in advanced zones of the pitch. Therefore, the aims of the present study were twofold: (1) to examine the defensive behaviors of soccer teams when gaining the ball in advanced zones of the pitch analyzing differences between them, and (2) to evaluate the effect of contextual variables on these defensive behaviors.

## Materials and Methods

### Match Sample

The sample was constituted by 1,095 defensive pieces of play initiated in the opposing half of the pitch obtained from 10 matches of the season 2010/11 of the Spanish La Liga (first division of the Spanish soccer league). The two competing teams of each match were analyzed, being a total of 13 teams in the sample. Three teams played at home twice and another one three times, whereas one team played away twice. The remaining eight teams only were analyzed once. From the 1,095 events, 1,062 were played in 11 against 11 conditions and the remaining 33 events were played with 11 players against 10. The final score of the games was two games ending 1-0, two ending 2-1, one ending 3-2, one ending 3-1, one ending 5-0, two ending 1-1, and one ending 1-2.

A defensive piece of play initiated in the opposing half was used as the basic unit of analysis. A defensive piece of play initiated in the opposing half starts when the attacking team initiates a possession of the ball in his own half of the pitch. To determine when the attacking team starts possession of the ball the next definition of Garganta (1997) was used. A team has possession of the ball when one of the next conditions is fulfilled:

• A player makes at least three consecutive touches to the ball.
• A pass is made.

A defensive piece of play initiated in the opposing half ends when defending teams gained possession of the ball after any of the above conditions occurs, as well as when:

• The attacking team delivers the ball out of the pitch.
• Any player of the attacking team is caught in offside position.
• Any player of the attacking team commits a foul whilst his team is in possession of the ball.
• A player of the attacking team makes a pass to a teammate who receives the ball behind the offside line and plays forward ending this piece of play in a shot or an entry into the defending team’s penalty area.

Variables used for this study are summarized in Table 1.

**TABLE 1:** Definitions of variables measured.

**Variable**	**Definition**
(1) Team analyzed (TA)	Name of the analyzed team.
(2) Match status (MS)	Score-line state of the analyzed team in this moment of the match: winning (W), drawing (D), or losing (L).
(3) Venue (V)	Team analyzed playing home (H) or away (A).
(4) Quality of opposition (QO)	Teams classified into any of the three groups according to their final ranking: 1^st^ to 6^th^ (1); 7^th^ to 13^th^ (2); 14^th^ to 20^th^ (3).
(5) Match period (MP)	Match period of the game divided in six periods of 15 min: 1–15 (1), 16–30 (2), 31–45 (3), 46–60 (4), 61–75 (5), 76–90 (6). In case that the defensive action occurred during the stoppage time of the first or second half of the game, it was assigned to the 31–45 or 76–90 period respectively.
(6) Distance from the least advanced outfield defender to his goal line (DLAODGL)	Distance from the least advanced outfield defender to his goal line when the analyzed team gained the ball or the attacking team made a pass to a player who received the ball behind the offside line and made a shot or entry into the penalty area. Registered once at the moment when the analyzed team gained the ball or the pass to the player who received the ball behind the offside line was made.
(7) Distance between the player in possession of the ball to the nearest defender (DPPBND)	Mean of the distance between the first three players in possession of the ball of the attacking team to their respective nearest defender. In the case that only one or two players had possession of the ball before losing it, one or two records were registered respectively to calculate that mean.
(8) Pass length (PL)	Length of the last pass that attacking teams made when conceded possession of the ball to the analyzed team. PL is defined as the distance from the last player of the attacking team who touched the ball to the player of the defending team who gained it. When attacking teams gave possession of the ball delivering it out of the pitch it was registered the distance from the last player who touched the ball to the ball the last 10^th^ of second before the ball left the pitch. This variable was not registered when defending teams gained the ball because any player of the attacking team was caught in offside position or committed a foul. Likewise, this variable was not registered when defensive pieces of play ended because a player of the attacking team made a pass to a teammate who received the ball behind the offside line and played forward ending this piece of play in a shot or an entry into the defending team’s penalty area.
(9) Pass number (PN)	Number of passes made by attacking teams before analyzed teams gained the ball.
(10) Duration (D)	Seconds elapsed from the beginning of the defensive piece of play initiated in the opposing half until it ended.
(11) Outcome of defensive pieces of play (ODPP)	11.1. Receiving a Dangerous Situation (RDS). The analyzed team received a shot or entry into the penalty area after an opponent had made a pass to a teammate who received the ball behind the offside line and played forward. The offside line was displayed by the Amisco Pro^®^ Animation Mode. An entry into the penalty area was defined as an event that takes place either when the team in possession of the ball passed it into the opponent’s penalty area (regardless of whether the pass was received by a teammate) or when a player in possession of the ball entered into that area of the pitch. Entry into the penalty area has been demonstrated to be a performance indicator that differentiates between winning and losing teams (Ruiz-Ruiz et al., 2013).
	11.2. Ball Gain Zone 1 (BGZ1). The pitch was divided into different zones according to the Amisco Pro^®^ system, that divides the pitch into six transversal zones parallel to the halfway and goal lines (see Figure 1). In the present study defensive pieces of play could start in any of the three zones of the attacking team’s half of the pitch: zone 4, zone 5, and zone 6. Zone 4 was the one closer to the halfway line and zone 6 was the one closer to the attacking team’s goal. For Ball Gain in Zone 1, the analyzed team gained the ball in zone 1. This is the zone closer to the defending team’s goal.
	11.3. Ball Gain Zone 2 (BGZ2). The analyzed team gained the ball in zone 2.
	11.4. Ball Gain Zone 3 (BGZ3). The analyzed team gained the ball in zone 3.
	11.5. Ball Gain Zone 4 (BGZ4). The analyzed team gained the ball in zone 4.
	11.6. Ball Gain Zone 5 (BGZ5). The analyzed team gained the ball in zone 5.
	11.7. Ball Gain Zone 6 (BGZ6). The analyzed team gained the ball in zone 6. This is the zone closer to the attacking team’s goal.
(12) Outcome of offensive pieces of play following ball gains (OOPPFBG)	This variable was only registered in those cases where defensive pieces of play ended on ball gains (11.2; 11.3; 11.4; 11.5; 11.6; 11.7).
	12.1 Giving Possession (GP). This event took place when after gaining the ball the analyzed team gave it to the opposition again.
	12.2. Creating a Dangerous Situation (CDS). After gaining the ball, the analyzed team made a shot or entry into the opposing penalty area.

### Data Collection Procedure

**FIGURE 1:**
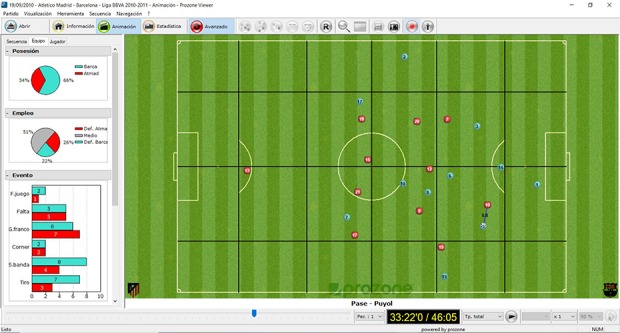
Animation Mode of the Amisco Pro^®^ system.

Sampled matches were registered using the semiautomatic tracking system Amisco Pro^®^. This match analysis system tracks the movements of the ball and every player during the whole match and creates a two-dimensional animated reconstruction of player movements that allow the analysis of teams and players. Information for every single match is stored on a DVD for post-game analysis, and a specific piece of software (Amisco Viewer) is used for data extraction (see Figure 1). The details of the Amisco Pro^®^ (Carling et al., 2008) and the accuracy and reliability of this system have been described elsewhere (Zubillaga, 2006; Randers et al., 2010).

Written permission from Amisco was obtained before the data collection. Ethics approval for all experimental procedures was granted by the Human Research Ethics Committee of the University of Granada. Two observers carried out the data collection. Prior to intra- and inter-observer reliability tests, the analysts underwent several sessions of analysis training in order to be familiar with the analysis system and its procedure. Subsequently, observers coded the same match to conduct the intra- and inter-observer reliability tests. Occurrence agreement (Gast, 2010) for defensive pieces of play initiated in the opposing half was 92%. Outcome of Defensive Pieces of Play was the only categorical variable and the kappa values obtained to assure the intra-observer reliability of the observation process were 0.90 and 0.91 for observer 1 and observer 2 respectively; and 0.79 for inter-observer reliability. These kappa values showed a ‘very good’ strength of agreement at the intra-observer level; and a ‘good’ strength of agreement at the inter-observer level (Altman, 1991). To assess the reliability of the continuous variables Distance from Least Advanced Outfield Defender to his Goal Line, Distance between the Player in Possession of the Ball to the Nearest Defender, Pass Length, Pass Number, and Duration; intra-class correlation (ICC), Pearson’s r, and Typical error of measurement (TEM) were calculated for each variable following the procedure according to Hopkins (2000, 2015) (Table 2).

**TABLE 2:** Reliability of continuous tactical variables.

**Variable**	**Mean ± SD**	**Intra-class correlation (ICC)**	**Pearson’s r**	**Typical error of measurement (TEM)**
DLAODGL	33.713.9	0.98	0.97	2.19
DPPBND	4.42.2	0.99	0.99	0.20
PL	21.718.3	0.99	0.99	0.86
PN	2.92.4	0.99	0.99	0.19
D	11.68.3	0.97	0.97	1.51

*DLAODGL, distance from least advanced outfield defender to his goal line; DPPBND, distance between the player in possession of the ball to the nearest defender; PL, pass length; PN, pass number; D, duration.*

### Statistical Analysis

Firstly, cross-tabulations were conducted between the categorical variables ‘team analyzed,’ ‘outcome of defensive pieces of play originating from the opposing half,’ and ‘outcome of offensive pieces of play following ball gains’ using Chi-square (χ^2^) tests and Cramer’s V statistic, in order to analyze the behavior of teams when gaining the ball in advanced zones of the pitch. To assess the effect of the contextual variables (i.e., match status, venue, quality of opposition, and match period) on gaining the ball in advanced zones of the pitch, additional cross-tabulations were conducted between each contextual variable and the variable ‘outcome of defensive pieces of play originating from the opposing half.’ A Cramer’s V effect size of φ_c_ = 0.1 was considered a small effect size; an effect size of φ_c_ = 0.3 was considered a medium effect size, while φ_c_ = 0.5 was considered a large effect size (Cohen, 1988). In addition, adjusted residuals were calculated to check relevant values from significant Chi-square tests (Field, 2017). Secondly, normality of the quantitative variables ‘distance from the least advanced outfield defender to his goal line when defending teams gained the ball,’ ‘duration of defensive pieces of play,’ ‘distance between the player in possession of the ball to the nearest defender,’ ‘length of the last pass that attacking teams made when conceded possession of the ball to defending teams,’ and ‘number of passes made before defending teams gained the ball’ was checked through visual exploration and Kolmogorov–Smirnoff tests. Although normality distribution could not be established for these variables, the assumption of normality was considered according to the Central Limit Theorem in such a large sample (*n* ≥ 30) (Field, 2017). Afterward, five one way-ANOVA tests were used with the five continuous variables mentioned previously as dependent variables, and the variable ‘team’ as the factor to examine the defensive behaviors of teams. Violations of homogeneity of variances were noted in the tests for the duration of defensive pieces of play, distance between the player in possession of the ball to the nearest defender and number of passes made before defending teams gained the ball. The Welch test was conducted for correction for unequal variances. Eta squared (η^2^) values are provided as a measure of effect size. An eta-squared effect size of η^2^ = 0.01 was considered a small effect size; an effect size of η^2^ = 0.06 was considered a medium effect size, while η^2^ = 0.14 was considered a large effect size (Cohen, 1988). Games-Howell *post hoc* were used to look at comparisons when interaction effects were found in those tests. For the variables with homogeneity of variances, LSD *post hoc* were used.

Thirdly, a two-step cluster analysis using log-likelihood as the distance measure and Akaike’s Information Criterion (AIC) as the clustering criterion (Burnham and Anderson, 2004) was conducted to group defensive variables on clusters that describe a general defensive behavior of teams when defending in the opposite half of the pitch, and to define match context where these defensive sequences occur. Defensive variables were considered as inputs and contextual variables were considered as evaluation fields in the model. Predictor importance showed the relative importance of each variable for estimating the model.

All the analyses were carried out with IBM SPSS Statistics for Windows, version 26 (IBM, Corp., Armonk, NY, United States). The alpha level for significance for all analyses was set at *p* < 0.05.

## Results

The relationship between team analyzed and outcome of defensive pieces of play originating from the opposing half was significant χ^2^(72, *N* = 1095) = 111.87, *p* < 0.01. The effect size was small to medium (Cramer’s *V* = 0.22). Adjusted residuals showed significant differences between teams in the zones where they gained the ball (Table 3). Firstly, Barcelona was the team that showed a higher likelihood of gaining possession of the ball in advanced zones of the pitch than the average. They gained seven more balls than the average in zone 5. Villareal showed a higher likelihood of gaining possession in zone 4; however they also showed a lower likelihood of gaining possession in zone 5. Secondly, there were 2 teams that were more likely to regain possession of the ball in zone 1, Bilbao and Levante, 7.9 and 9.3 more balls than the average respectively. Finally, Barcelona, Bilbao and Zaragoza were the teams that were less likely to receive a dangerous situation. On the other hand, Valencia and Hercules were the ones with a higher likelihood of receiving a dangerous situation. The relationship between team analyzed and outcome of offensive pieces of play following ball gains was significant χ^2^(12, *N* = 1030) = 49.92, *p* < 0.001. The effect size was small to medium (Cramer’s *V* = 0.22). Valencia and Hercules were the teams that created more dangerous situations than the average; and Bilbao, Osasuna, and Mallorca the teams that conceded possession to the opposite team more times than average (Table 4).

**TABLE 3:** Cross-tabulation of outcome of defensive pieces of play (ODPP) and teams analyzed.

	**ODPP**							
**Team**		**RDS**	**BGZ1**	**BGZ2**	**BGZ3**	**BGZ4**	**BGZ5**	**BGZ6**
Barcelona	Count	0	10	18	18	13	13	1
	Expected count	3.9	11.5	17.5	19.7	12.8	6	1.5
	% Within ODPP	0	5.8	6.9	6.1	6.8	14.4	4.3
	Adjusted residuals	−2.1	−0.5	−0.2	−0.5	0.1	3.1	−0.5
Real Madrid	Count	2	5	15	15	14	4	2
	Expected count	3.1	9	13.6	15.4	10	4.7	1.2
	% Within ODPP	3.4	2.9	5.7	5.1	7.3	4.4	8.7
	Adjusted residuals	−0.6	−1.5	0.4	−0.1	1.4	−0.3	0.8
Valencia	Count	11	13	24	33	21	12	1
	Expected count	6.2	18.2	27.5	31.1	20.2	9.5	2.4
	% Within ODPP	18.6	7.5	9.2	11.1	10.9	13.3	4.3
	Adjusted residuals	2.1	−1.4	−0.8	0.4	0.2	0.9	−1
Villareal	Count	5	3	10	12	14	0	2
	Expected count	2.5	7.3	11	12.4	8.1	3.8	1
	% Within ODPP	8.5	1.7	3.8	4.1	7.3	0	8.7
	Adjusted residuals	1.7	−1.8	−0.4	−0.1	2.4	−2.1	1.1
Bilbao	Count	1	24	19	27	24	6	1
	Expected count	5.5	16.1	24.4	27.6	17.9	8.4	2.1
	% Within ODPP	1.7	13.9	7.3	9.1	12.5	6.7	4.3
	Adjusted residuals	−2.1	2.2	−1.3	−0.1	1.7	−0.9	−0.8
Atletico Madrid	Count	15	35	61	67	31	17	6
	Expected count	12.5	36.7	55.5	62.7	40.7	19.1	4.9
	% Within ODPP	25.4	20.2	23.3	22.6	16.1	18.9	26.1
	Adjusted residuals	0.8	−0.3	1	0.7	−1.9	−0.6	0.6
Osasuna	Count	3	15	23	22	9	7	1
	Expected count	4.3	12.6	19.1	21.6	14	6.6	1.7
	% Within ODPP	5.1	8.7	8.8	7.4	4.7	7.8	4.3
	Adjusted residuals	−0.7	0.8	1.1	0.1	−1.5	0.2	−0.6
Zaragoza	Count	0	12	14	20	18	6	1
	Expected count	3.8	11.2	17	19.2	12.4	5.8	1.5
	% Within ODPP	0	6.9	5.3	6.8	9.4	6.7	4.3
	Adjusted residuals	−2.1	0.3	−0.9	0.2	1.8	0.1	−0.4
Levante	Count	0	18	17	10	6	3	1
	Expected count	3	8.7	13.2	14.9	9.6	4.5	1.2
	% Within ODPP	0	10.4	6.5	3.4	3.1	3.3	4.3
	Adjusted residuals	−1.8	3.5	1.2	−1.5	−1.3	−0.8	−0.1
Real Sociedad	Count	8	11	23	37	19	12	3
	Expected count	6.1	17.9	27	30.5	19.8	9.3	2.4
	% Within ODPP	13.6	6.4	8.8	12.5	9.9	13.3	13
	Adjusted residuals	0.8	−1.9	−0.9	1.4	−0.2	1	0.4
Getafe	Count	3	11	12	17	9	2	0
	Expected count	2.9	8.5	12.9	14.6	9.5	4.4	1.1
	% Within ODPP	5.1	6.4	4.6	5.7	4.7	2.2	0
	Adjusted residuals	0.1	0.9	−0.3	0.8	−0.2	−1.2	−1.1
Mallorca	Count	2	7	12	5	4	3	1
	Expected count	1.8	5.4	8.1	9.2	6	2.8	0.7
	% Within ODPP	3.4	4	4.6	1.7	2.1	3.3	4.3
	Adjusted residuals	0.1	0.8	1.6	−1.6	−0.9	0.1	0.3
Hercules	Count	9	9	14	13	10	5	3
	Expected count	3.4	10	15.1	17	11	5.2	1.3
	% Within ODPP	15.3	5.2	5.3	4.4	5.2	5.6	13
	Adjusted residuals	3.2	−0.3	−0.3	−1.2	−0.4	−0.1	1.5

*RDS, receiving a dangerous situation; BGZ1, ball gain in zone 1; BGZ2, ball gain in zone 2; BGZ3, ball gain in zone 3; BGZ4, ball gain in zone 4; BGZ5, ball gain in zone 5; BGZ6, ball gain in zone 6.*

**TABLE 4:** Cross-tabulation of offensive outcome of pieces of play following ball gains (OOPPFBG) and teams analyzed.

		**OOPPFBG**	
**Team**		**CDS**	**GP**
Barcelona	Count	15	58
	Expected count	13.9	59.1
	% Within OOPPFBG	7.7	7.0
	Adjusted residuals	0.3	−0.3
Real Madrid	Count	14	41
	Expected count	10.5	44.5
	% Within OOPPFBG	7.1	4.9
	Adjusted residuals	1.2	−1.2
Valencia	Count	36	68
	Expected count	19.8	84.2
	% Within OOPPFBG	18.4	8.2
	Adjusted residuals	4.3	−4.3
Villareal	Count	9	32
	Expected count	7.8	33.2
	% Within OOPPFBG	4.6	3.8
	Adjusted residuals	0.5	−0.5
Bilbao	Count	6	95
	Expected count	19.2	81.8
	% Within OOPPFBG	3.1	11.4
	Adjusted residuals	−3.5	3.5
Atletico Madrid	Count	39	175
	Expected count	40.7	173.3
	% Within OOPPFBG	19.9	21.0
	Adjusted residuals	−0.3	0.3
Osasuna	Count	8	69
	Expected count	14.7	62.3
	% Within OOPPFBG	4.1	8.3
	Adjusted residuals	−2.0	2.0
Zaragoza	Count	14	54
	Expected count	12.9	55.1
	% Within OOPPFBG	7.1	6.5
	Adjusted residuals	0.3	−0.3
Levante	Count	9	46
	Expected count	10.5	44.5
	% Within OOPPFBG	4.6	5.5
	Adjusted residuals	−0.5	0.5
Real Sociedad	Count	20	85
	Expected count	20.0	85.0
	% Within OOPPFBG	10.2	10.2
	Adjusted residuals	0	0
Getafe	Count	6	45
	Expected count	9.7	41.3
	% Within OOPPFBG	3.1	5.4
	Adjusted residuals	−1.4	1.4
Mallorca	Count	1	31
	Expected count	6.1	25.9
	% Within OOPPFBG	0.5	3.7
	Adjusted residuals	−2.3	2.3
Hercules	Count	19	35
	Expected count	10.3	43.7
	% Within OOPPFBG	9.7	4.2
	Adjusted residuals	3.1	−3.1

*CDS, creating a dangerous situation; GP, giving possession.*

The results for contextual variables showed that the relationship between outcome of defensive pieces of play originating from the opposing half and match status (winning, drawing or losing) was significant: χ^2^(12, *N* = 1095) = 25.87, *p* < 0.05. The effect size was small to medium (Cramer’s *V* = 0.11). Results showed that teams gained the ball significantly more times in zone 1 when winning; and when they were losing, gained the ball significantly fewer times in zones 1 and 2, and more times in zone 5 (Table 5). The relationship between outcome of defensive pieces of play originating from the opposing half and venue (playing at home or away) was not significant: χ^2^(6, *N* = 1095) = 5.95, *p* > 0.05. The relationship between outcome of defensive pieces of play originating from the opposing half and quality of opposition was significant: χ^2^(12, *N* = 1095) = 21.19, *p* < 0.05. The effect size was small (Cramer’s *V* = 0.10). Teams gained the ball significantly more times in zone 2 and fewer times in zones 3 and 4 when playing against strong opposition, and significantly gained the ball fewer times in zone 2 and more times in zone 3 when playing a weak opposition (Table 6). The relationship between outcome of defensive pieces of play originating from the opposing half and match period was not significant: χ^2^(30, *N* = 1095) = 38.53, *p* > 0.05.

**TABLE 5:** Cross-tabulation of outcome of defensive pieces of play (ODPP) and match status.

**ODPP**		**MS**		
		**Winning**	**Drawing**	**Losing**
RDS	Count	18	21	20
	Expected count	19.8	18.3	20.9
	% Within match status	4.9	6.2	5.2
	Adjusted residual	−0.5	0.8	−0.2
BGZ1	Count	76	48	49
	Expected count	58.1	53.7	61.1
	% Within match status	20.7	14.	12.7%
	Adjusted residual	3.1	−1	−2.1
BGZ2	Count	92	92	78
	Expected count	88.1	81.4	92.6
	% Within match status	25.0	27.1	20.2
	Adjusted residual	0.6	1.6	−2.2
BGZ3	Count	94	93	109
	Expected count	99.5	91.9	104.6
	% Within match status	25.5	27.4	28.2
	Adjusted residual	−0.8	0.2	0.6
BGZ4	Count	54	60	78
	Expected count	64.5	59.6	67.9
	% Within match status	14.7	17.6	20.2
	Adjusted residual	−1.8	0.1	1.7
BGZ5	Count	30	19	41
	Expected count	30.2	27.9	31.8
	% Within match status	8.2	5.6	10.6
	Adjusted residual	−0.1	−2.1	2.1
BGZ6	Count	4	7	12
	Expected count	7.7	7.1	8.1
	% Within match status	1.1	2.1	3.1
	Adjusted residual	−1.7	−0.1	1.7

*RDS, receiving a dangerous situation; BGZ1, ball gain in zone 1; BGZ2, ball gain in zone 2; BGZ3, ball gain in zone 3; BGZ4, ball gain in zone 4; BGZ5, ball gain in zone 5; BGZ6, ball gain in zone 6.*

**TABLE 6:** Cross-tabulation of outcome of defensive pieces of play (ODPP) and quality of opposition.

**ODPP**		**Quality of opposition**		
		**1**	**2**	**3**
RDS	Count	28	17	14
	Expected count	23.8	18.8	16.4
	% Within quality of opposition	6.3	4.9	4.6
	Adjusted residual	1.1	−0.5	−0.7
BGZ1	Count	78	48	47
	Expected count	69.8	55.1	48.0
	% Within quality of opposition	17.6	13.8	15.5
	Adjusted residual	1.4	−1.3	−0.2
BGZ2	Count	122	84	56
	Expected count	105.8	83.5	72.7
	% Within quality of opposition	27.6	24.1	18.4
	Adjusted residual	2.3	0.1	−2.6
BGZ3	Count	104	93	99
	Expected count	119.5	94.3	82.2
	% Within quality of opposition	23.5	26.6	32.6
	Adjusted residual	−2.1	−0.2	2.6
BGZ4	Count	64	66	62
	Expected count	77.5	61.2	53.3
	% Within quality of opposition	14.5	18.9	20.4
	Adjusted residual	−2.2	0.8	1.5
BGZ5	Count	36	32	22
	Expected count	36.3	28.7	25.0
	% Within quality of opposition	8.1	9.2	7.2
	Adjusted residual	−0.1	0.8	−0.7
BGZ6	Count	10	9	4
	Expected count	9.3	7.3	6.4
	% Within quality of opposition	2.3	2.6	1.3
	Adjusted residual	0.3	0.8	−1.1

*RDS, receiving a dangerous situation; BGZ1, ball gain in zone 1; BGZ2, ball gain in zone 2; BGZ3, ball gain in zone 3; BGZ4, ball gain in zone 4; BGZ5, ball gain in zone 5; BGZ6, ball gain in zone 6.*

**FIGURE 2:**
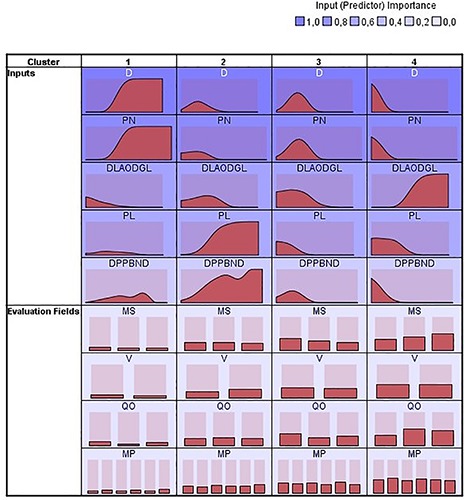
Relative distributions of variables for each cluster. DLAODGL, distance from least advanced outfield defender to his goal line; DPPBND, distance between the player in possession of the ball to the nearest defender; PL, pass length; PN, pass number; D, duration; MS, match status; V, venue; QO, quality of opposition; MP, match period.

**FIGURE 3:**
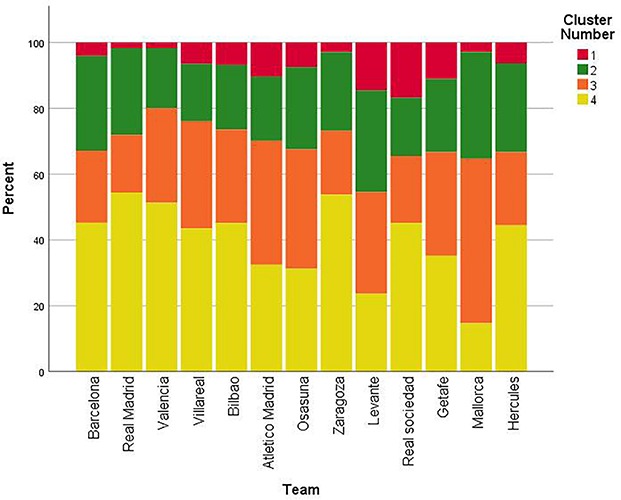
Percentage of defensive pieces of play for each cluster used by teams. Cluster 1, defense close to the own goal; Cluster 2, mid-positioning defense on the pitch using a less intense pressure to attacking players; Cluster 3, mid-positioning defense on the pitch using a more intense pressure to attacking players; Cluster 4, high-pressure defense in advanced zones of the pitch.

There were significant differences among teams in the distance from the least advanced outfield defender to his goal line, *F*(12,1082) = 5.31, *p* < 0.001, η^2^ = 0.06 (Tables 7, 8); in duration of defensive pieces of play initiated in the opposing half of the pitch, *F*(12,1082) = 4.22, *p* < 0.001, η^2^ = 0.05 (Tables 7, 9); in distance between the player in possession of the ball to the nearest defender in defensive pieces of play initiated in the opposing half of the pitch, *F*(12,1081) = 2.68, *p* < 0.005, η^2^ = 0.03 (Tables 7, 10); and in number of passes made by attacking teams before defending teams gained the ball in defensive pieces of play initiated in the opposing half of the pitch, *F*(12,1082) = 3.50, *p* < 0.001, η^2^ = 0.04 (Tables 7, 11). On the other hand, there were no significant differences among teams in length of the pass that attacking teams made when conceded possession of the ball to defending teams in defensive pieces of play initiated in the opposing half of the pitch, *F*(12,1078) = 0.76, *p* > 0.05, η^2^ = 0.01 (Table 7). Cluster analysis revealed four groups according to the defensive variables (i.e., distance from least advanced outfield defender to his goal line, distance between the player in possession of the ball to the nearest defender, pass length, pass number and duration) and the contextual variables (i.e., match status, venue, quality of opposition, and match period). Clusters 1, 2, 3, and 4 comprised the 7.9, 22.3, 29.4, and 40.5% of the sample size respectively. The AIC value of the cluster analysis modeling the four clusters was 2,072.32. The relative distribution of variables for each cluster and their respective Predictor Importance (PI) is shown in Figure 2. The cluster analysis showed the following PI values for defensive variables in descendant order; duration (PI = 1), pass number (PI = 0.88), distance from least advanced outfield defender to his goal line (PI = 0.65), pass length (PI = 0.59), distance between the player in possession of the ball to the nearest defender (PI = 0.25). In addition, it also showed that PI attributed to contextual variables was very low; quality of opposition (PI = 0.02) match status (PI = 0.01), venue (PI < 0.01), match period (PI < 0.01). Figure 3 presents the percentage of defensive pieces of play associated with each cluster used by teams.

**TABLE 7:** Means and standard deviations of tactical variables displayed by teams in defensive pieces of play initiated in the opposing half of the pitch.

**Team**	**DLAODGL (meters)**	**D (seconds)**	**DPPBND (meters)**	**PL (meters)**	**PN**
Barcelona	34.19 (14.57)	9.60 (6.70)	4.52 (4.01)	19.53 (18.11)	2.36 (1.87)
Real Madrid	37.93 (12.19)	8.40 (5.63)	3.26 (1.45)	21.48 (18.37)	2.11 (1.73)
Valencia	35.48 (13.09)	10.67 (7.79)	4.33 (2.84)	16.43 (15.31)	2.58 (2.31)
Villareal	36.08 (11.69)	11.97 (8.5)	4.00 (2.57)	16.60 (16.01)	3.11 (2.58)
Bilbao	31.45 (14.60)	10.72 (7.15)	3.85 (1.83)	19.95 (18.07)	2.78 (2.25)
Atletico Madrid	30.36 (13.55)	13.41 (8.82)	4.60 (2.71)	16.99 (16.97)	3.56 (3.02)
Osasuna	29.72 (14.09)	14.22 (9.12)	5.20 (2.88)	19.04 (18.04)	3.50 (2.65)
Zaragoza	33.87 (12.10)	9.61 (6.59)	4.99 (3.77)	17.23 (15.75)	2.35 (1.94)
Levante	25.53 (15.81)	14.05 (8.46)	5.50 (4.00)	19.49 (14.97)	3.55 (2.42)
Real Sociedad	34.53 (11.66)	13.57 (11.93)	4.93 (3.71)	17.91 (17.23)	3.75 (3.89)
Getafe	29.57 (12.81)	14.51 (11.06)	4.99 (3.14)	21.65 (20.82)	3.48 (3.44)
Mallorca	24.25 (13.12)	12.13 (6.59)	4.81 (2.98)	19.08 (21.28)	2.56 (1.61)
Hercules	34.17 (13.69)	10.84 (7.81)	4.66 (2.32)	18.51 (17.65)	2.81 (2.85)

*DLAODGL, distance from the least advanced outfield defender to his goal line; D, duration of defensive pieces of play initiated in the opposing half; DPPBND, distance between the player in possession of the ball to the nearest defender; PL, length of the last pass that attacking teams made when conceded possession of the ball to the analyzed team; PN, number of passes made by attacking teams before analyzed teams gained the ball.*

**TABLE 8:** LSD multiple comparisons for distance from the least advanced outfield defender to his goal line (DLAODGL) in defensive pieces of play initiated in the opposing half across the 13 teams analyzed.

**Team**	**Barcelona**	**Real Madrid**	**Valencia**	**Villareal**	**Bilbao**	**Atletico Madrid**	**Osasuna**	**Zaragoza**	**Levante**	**Real Sociedad**	**Getafe**	**Mallorca**
Barcelona												
Real Madrid	N.S.											
Valencia	N.S.	N.S.										
Villareal	N.S.	N.S.	N.S.									
Bilbao	N.S.	*p* < 0.01	*p* < 0.05	N.S.								
Atletico Madrid	*p* < 0.05	*p* < 0.001	*p* < 0.001	*p* < 0.01	N.S.							
Osasuna	*p* < 0.05	*p* < 0.001	*p* < 0.01	*p* < 0.05	N.S.	N.S.						
Zaragoza	N.S.	N.S.	N.S.	N.S.	N.S.	N.S.	N.S.					
Levante	*p* < 0.001	*p* < 0.001	*p* < 0.001	*p* < 0.001	*p* < 0.001	*p* < 0.05	N.S.	*p* < 0.01				
Real Sociedad	N.S.	N.S.	N.S.	N.S.	N.S.	*p* < 0.01	*p* < 0.05	N.S.	*p* < 0.001			
Getafe	N.S.	*p* < 0.01	*p* < 0.01	*p* < 0.05	N.S.	N.S.	N.S.	N.S.	N.S.	*p* < 0.05		
Mallorca	*p* < 0.001	*p* < 0.001	*p* < 0.001	*p* < 0.001	*p* < 0.01	*p* < 0.05	*p* < 0.05	*p* < 0.01	N.S.	*p* < 0.001	N.S.	
Hercules	N.S.	N.S.	N.S.	N.S.	N.S.	*p* < 0.05	*p* < 0.05	N.S.	*p* < 0.001	N.S.	N.S.	*p* < 0.005

*N.S., no significant difference.*

**TABLE 9:** Games-Howell multiple comparisons for duration (D) in defensive pieces of play initiated in the opposing half across the 13 teams analyzed.

**Team**	**Barcelona**	**Real Madrid**	**Valencia**	**Villareal**	**Bilbao**	**Atletico Madrid**	**Osasuna**	**Zaragoza**	**Levante**	**Real Sociedad**	**Getafe**	**Mallorca**
Barcelona												
Real Madrid	N.S.											
Valencia	N.S.	N.S.										
Villareal	N.S.	N.S.	N.S.									
Bilbao	N.S.	*p* < 0.01	N.S.	N.S.								
Atletico Madrid	*p* < 0.01	*p* < 0.001	N.S.	N.S.	N.S.							
Osasuna	*p* < 0.05	*p* < 0.01	N.S.	N.S.	N.S.	N.S.						
Zaragoza	N.S.	N.S.	N.S.	N.S.	N.S.	*p* < 0.01	*p* < 0.05					
Levante	N.S.	*p* < 0.01	N.S.	N.S.	N.S.	N.S.	N.S.	N.S.				
Real Sociedad	N.S.	*p* < 0.05	N.S.	N.S.	N.S.	N.S.	N.S.	N.S.	N.S.			
Getafe	N.S.	*p* < 0.05	N.S.	N.S.	N.S.	N.S.	N.S.	N.S.	N.S.	N.S.		
Mallorca	N.S.	N.S.	N.S.	N.S.	N.S.	N.S.	N.S.	N.S.	N.S.	N.S.	N.S.	
Hercules	N.S.	N.S.	N.S.	N.S.	N.S.	N.S.	N.S.	N.S.	N.S.	N.S.	N.S.	N.S.

*N.S., no significant difference.*

**TABLE 10:** Games-Howell multiple comparisons for distance between the player in possession of the ball to the nearest defender (DPPBND) in defensive pieces of play initiated in the opposing half across the 13 teams analyzed.

**Team**	**Barcelona**	**Real Madrid**	**Valencia**	**Villareal**	**Bilbao**	**Atletico Madrid**	**Osasuna**	**Zaragoza**	**Levante**	**Real Sociedad**	**Getafe**	**Mallorca**
Barcelona												
Real Madrid	N.S.											
Valencia	N.S.	N.S.										
Villareal	N.S.	N.S.	N.S.									
Bilbao	N.S.	N.S.	N.S.	N.S.								
Atletico Madrid	N.S.	*p* < 0.001	N.S.	N.S.	N.S.							
Osasuna	N.S.	*p* < 0.001	N.S.	N.S.	*p* < 0.05	N.S.						
Zaragoza	N.S.	*p* < 0.05	N.S.	N.S.	N.S.	N.S.	N.S.					
Levante	N.S.	*p* < 0.05	N.S.	N.S.	N.S.	N.S.	N.S.	N.S.				
Real Sociedad	N.S.	*p* < 0.01	N.S.	N.S.	N.S.	N.S.	N.S.	N.S.	N.S.			
Getafe	N.S.	*p* < 0.05	N.S.	N.S.	N.S.	N.S.	N.S.	N.S.	N.S.	N.S.		
Mallorca	N.S.	N.S.	N.S.	N.S.	N.S.	N.S.	N.S.	N.S.	N.S.	N.S.	N.S.	
Hercules	N.S.	*p* < 0.05	N.S.	N.S.	N.S.	N.S.	N.S.	N.S.	N.S.	N.S.	N.S.	N.S.

*N.S., no significant difference.*

**TABLE 11:** Games-Howell multiple comparisons for number of passes made before defending teams gained the ball (PN) in defensive pieces of play initiated in the opposing half across the 13 teams analyzed.

**Team**	**Barcelona**	**Real Madrid**	**Valencia**	**Villareal**	**Bilbao**	**Atletico Madrid**	**Osasuna**	**Zaragoza**	**Levante**	**Real Sociedad**	**Getafe**	**Mallorca**
Barcelona												
Real Madrid	N.S.											
Valencia	N.S.	N.S.										
Villareal	N.S.	N.S.	N.S.									
Bilbao	N.S.	N.S.	N.S.	N.S.								
Atletico Madrid	*p* < 0.01	*p* < 0.001	N.S.	N.S.	N.S.							
Osasuna	N.S.	*p* < 0.05	N.S.	N.S.	N.S.	N.S.						
Zaragoza	N.S.	N.S.	N.S.	N.S.	N.S.	*p* < 0.01	N.S.					
Levante	N.S.	*p* < 0.05	N.S.	N.S.	N.S.	N.S.	N.S.	N.S.				
Real Sociedad	N.S.	*p* < 0.05	N.S.	N.S.	N.S.	N.S.	N.S.	N.S.	N.S.			
Getafe	N.S.	N.S.	N.S.	N.S.	N.S.	N.S.	N.S.	N.S.	N.S.	N.S.		
Mallorca	N.S.	N.S.	N.S.	N.S.	N.S.	N.S.	N.S.	N.S.	N.S.	N.S.	N.S.	
Hercules	N.S.	N.S.	N.S.	N.S.	N.S.	N.S.	N.S.	N.S.	N.S.	N.S.	N.S.	N.S.

*N.S., no significant difference.*

## Discussion

The aims of this study were (1) to examine the defensive behaviors of soccer teams when gaining the ball in advanced zones of the pitch analyzing differences between them, and (2) to evaluate the effect of contextual variables on these defensive behaviors. Differences between teams of defensive behaviors during defensive pieces of play initiated in the opposite half of the pitch were revealed by the cross-tabulations and cluster analysis. In addition, two of the contextual variables analyzed, match status and quality of opposition seemed to influence the defensive behavior of soccer teams.

The findings of the present study demonstrated that teams employed different defensive behaviors in defensive pieces of play initiated in the opposing half of the pitch. According to the defensive variables represented in clusters, we could consider that cluster 1 represented a defense close to the own goal (high ‘duration’ and ‘number of passes,’ and low ‘distance from least advanced outfield defender to his goal line’); cluster 2 represented a mid-positioning defense on the pitch and providing a less intense pressure to attacking players (high ‘pass length’ and ‘distance between the player in possession of the ball to the nearest defender,’ and a middle ‘duration,’ ‘number of passes’ and ‘distance from least advanced outfield defender to his goal line’); cluster 3 represented similarly a mid-positioning defense whereas using a more intense pressure (middle ‘duration,’ ‘number of passes’ and ‘distance from least advanced outfield defender to his goal line,’ and low ‘pass length’ and ‘distance between the player in possession of the ball to the nearest defender’); and cluster 4 represented a high-pressure defense (high ‘distance from least advanced outfield defender to his goal line,’ and low ‘duration,’ ‘number of passes,’ ‘pass length,’ and ‘distance between the player in possession of the ball to the nearest defender’). Teams differed between each other in the defensive variables measured and the results provided a more detailed analysis of defensive behaviors in comparison with studies that only measured ball regains to define defensive team tactics. For example, our results showed that Barcelona was the team that gained the ball in advanced zones of the pitch significantly more times in comparison to other teams, therefore demonstrating their preference for using high pressure. These findings are in line with previous research that reported these tactics for Barcelona using network metrics (Buldu et al., 2019). In addition, general defensive behaviors used by teams based on the defensive variables analyzed could be considered defensive styles of play in line with previous research (Fernandez-Navarro et al., 2016), as this concept comprises the connection of several variables to describe a general behavior. The differences between teams suggest that they were specialized in the use of concrete defensive tactics, possibly due to the characteristics of their players (Clemente et al., 2013); or that teams changed their defensive behavior according to the contextual variables in soccer match-play (Fernandez-Navarro et al., 2018).

The importance of match status when analyzing soccer data have been highlighted in other research (Lupo and Tessitore, 2016). The results of this study showed, in line with previous findings (Almeida et al., 2014), that match status had an effect on the outcome of defensive pieces of play. When teams were winning, they gained more balls in the zone close to their own goal, suggesting that teams tended to use a low-pressure defense to maintain the scoreline. In the same way, several studies (Vogelbein et al., 2014; Fernandez-Navarro et al., 2018) reported the same behavior in these conditions during the game. Consequently, a possible reason for this would be the preference of the teams to defend close to their own goal to maintain their winning status and use direct or counterattack actions after gaining the ball afterward. This combination of low-pressure defense and direct/counterattacking play seems to be a reasonable tactic as players would be best positioned in defense to conduct the following direct/counterattacking play, taking advantage of the advanced position of the opposite team at that moment (Fernandez-Navarro et al., 2018). In contrast, teams gained more balls in advanced zones of the pitch when they were losing. This is in support of previous research (Almeida et al., 2014; Santos et al., 2017) that found an increase in ball recovery location when the teams were losing. One explanation for this behavior could be that teams tried to gain the ball as soon as possible in order to have more attacking chances to change the scoreline. As a matter of fact, Tenga and Larsen (2003) reported that a high-pressure play entailed more attacks that start in the attacking third, and would explain the use of this tactic. With respect to quality of opposition, the greater the quality of the opponent the lesser the chance of gaining the ball in advanced zones of the pitch. When teams played against strong opposition, significantly fewer balls were gained in zones 3 and 4 and more balls were gained in zone 2; whereas when teams faced a weak opposition, more balls were gained in zone 3 and fewer balls were gained in zone 2. Previous research also found the same tendency that playing against a strong opposition decrease the position of the defensive line on the pitch (Santos et al., 2017), and decreased the ball gains in more advanced positions of the pitch (Almeida et al., 2014). Similarly, Fernandez-Navarro et al. (2018) found that when teams played against strong opposition there was a decrease in the use of the high-pressure style of play. The fact that better-ranked teams have better players could perhaps be the reason for these teams applying this aggressive tactic in defense that requires intense efforts and good player positioning.

The data showed that there was no effect of venue on the defensive variables analyzed. The study by Gomez et al. (2012) supports these findings as they presented similar results. However, this contrast with the results of previous research reporting that teams playing home used to defend in more advanced zones of the pitch (Almeida et al., 2014; Santos et al., 2017), and increase the use of a high-pressure style of play (Fernandez-Navarro et al., 2018). This contradictory evidence could be caused by the different samples and methodologies used in research. Thus, further studies are needed to shed light on the effect of match location in defensive tactical aspects in soccer. In addition, the results of the present study showed no effect of match period on defensive pieces of play initiated in the opposite half of the pitch. Although other studies have evaluated match period for substitutions during a game (Gomez et al., 2016), or goal-scoring (Lago-Peñas et al., 2016; Pratas et al., 2016); no previous research reported the effect of this contextual variable on defensive actions in soccer.

A limitation of the present study is that only a sample of 10 matches was available for the study. It is possible that a larger set of matches might have reduced the variability of the analyzed sample. Ideally, the entire sample of matches in the league should have been used in order to allow a better generalization of the results. A second limitation is the high standard deviation in all the variables that indicates that data is spread out over a large range of values. This means that the conclusions of the present study should be taken carefully. Significant differences were found in tactical variables across the two independent variables outcome of defensive pieces of play initiated in the opposing half and team analyzed. However, high standard deviations show that different defensive performances were found in each level of the mentioned independent variables. This dispersion of the data reflects the chaotic nature of football. For practical applications, coaches and practitioners could use this performance profiling to evaluate the defensive behavior of the own team or the opposition, in order to prepare the teams better for competition. Furthermore, practitioners should pay attention to the contextual variables that affect the mentioned defensive behaviors when analyzing match performance. Future studies should consider contextual variables when analyzing match data, as it has been proved that they affect the tactical behavior of soccer teams.

## Conclusion

The analysis of the defensive variables revealed that teams employed different defensive tactical behaviors in competition, from high-pressure to a defense close to their own goal. Match status and quality of opposition were the contextual variables that influenced defensive pieces of play initiated in the opposite half of the pitch. These results provide a better understanding of the defensive behaviors of soccer teams during match-play.

## Data Availability Statement

The datasets generated for this study are available on request to the corresponding author.

## Author Contributions

JF-N: manuscript writing, review, data analysis, and editing. CR-R: data acquisition and coding, data analysis, and draft writing. AZ: study designing, data analysis, and supervision. LF: study designing and supervision.

## Conflict of Interest

The authors declare that the research was conducted in the absence of any commercial or financial relationships that could be construed as a potential conflict of interest.
